# Supplementary material for: Exploring the Determinants of Polypharmacy Prescribing and Dispensing Behaviors in Primary Care for the Elderly—Qualitative Study
Source: Int J Environ Res Public Health. 2023 Jan 12;20(2):1389. doi: 10.3390/ijerph20021389 (PMC9859068; doi:10.3390/ijerph20021389)
Supplement: Supplementary file 1 [file ijerph-20-01389-s001.zip › ijerph-2000403-supplementary.pdf]

TDF domains	Determinants (barriers and/or facilitators)	Illustrative quotes translated into English
**Knowledge**	• knowledge acquired in initial or continuing training (facilitator)<br>• knowledge of geriatric therapeutics (barrier or facilitator)<br>• Consulting/receiving recommendations (facilitator)<br>• Subscription to the journal "Prescrire" (facilitator)<br>• Using databases and software (facilitator)	"... I rely on initial training and continuing education especially..." (MG3)<br>"It's true that we don't necessarily have a lot of training on the geriatric side knowing that geriatrics at the therapeutic level is particular." (MG2)<br>"...yes (we receive recommendations) for example on antihypertensive drugs to be used in the elderly etc... yes we try, yes. The benzo etc...try to give hypnotics or short half-lives, yes, we try." (MG6)<br>"I subscribe to the journal Prescrire and I'm interested in it. Prescrire also criticizes all drugs...the only information I get from Prescrire..." (MG4)<br>"I look at the Vidal for the indication, the type of drug if I really don't know it and for what indication it can be prescribed. After that, I sometimes look on the internet for the indications of therapeutics..." (MG2)<br>"...afterwards, we have our professional software that will at least indicate interactions..." (PH3)
**Skills**	• Patient profile: helping or opposing (barrier or facilitator)<br>• Lack of specific training on communication skills and how to approach patients in this situation (barrier)<br>• Trusting relationship GPs, Pharmacist/Patient: relational aspect, interactions, explanations, shared decision (facilitator)<br>• Difficulties in communication between prescribers (specialists among themselves, GPs and specialists), between doctors and pharmacists (barrier)<br>• functioning in a network of professionals (facilitator)	"There are several patient profiles; there are those who as soon as you remove something it is "ah! I miss the tablet such and such ..." and there you are off for a big explanation of why you decided to stop it and just that you can be sure that the next month she will say "no, you have to put it back because it really is too late". etc." and then you have to explain why you decided to stop it and you can be sure that the following month she will say "no, you have to put it back because it really isn't working", so it is an obstacle for the patient... There are two profiles, one helping and one opposing, which are clearly encountered in practice." (MG1)<br>"...I think that we are not taught enough about that () that's for sure! ...I think that it's not worked on enough that's for sure, we are just told it's good it's not good and everything... yes but we have to manage to convince them... (training would be useful) yes, at least try to make us work to have a different approach. Maybe if we do it differently, if we tell them differently, maybe we can get a better result. '" (MG6)<br>"...Untrained yes, afterwards we learn on the job. " (MG5)<br>"... (specific training) it can help yes, if only to make sure the person understands the treatment.." (PH3)<br>"...it's always a listening an adaptation but I know that there are people with whom it's complicated to reduce prescriptions ... yes necessarily there is a more individual and psychological approach related to the relational and interaction that there is during a consultation. " (MG5)<br>"...I try to explain every time. It's a lot of discussion. Some people feel like we're trying to take something away from them, but if we explain why there's an interruption, it passes without a problem. " (MG2)<br>"...When people don't know us, when the relationship of trust is not necessarily established or we are at the beginning of establishing it, it absolutely implies particular communication techniques and notably shared decision making. "(MG3)<br>"...we try to communicate, it's more or less easy...we hardly interface with other prescribers unless we really have a big concern but otherwise not. "(MG1)<br>"...It's true that afterwards with other professionals there are times ... for example, I referred a patient with respiratory insufficiency and a history of coronary artery disease for the management of a recently discovered diabetes problem because it seemed to me that it wasn't necessarily wise to start with Metphormine and the diabetologist suggested metformin. So I have more difficulty with other professionals..." (MG5)<br>"...in town medicine it is still local correspondents with whom we work regularly so we call each other easily and there is no problem. " (MG2)<br>"...If a prescription is a bit unusual, knowing the prescribing colleague, I call him directly. "(MG3)

TDF domains	Determinants (barriers and/or facilitators)	Illustrative quotes translated into English
Social/ professional role and identity	• GPs conscious or not of their pivotal/oversight role (barrier or facilitator)<br>• Pharmacists' belief in their firewall role (in tandem with the pivotal role of doctors) (facilitator)<br>• GPs' belief in the complementary role (more or less effective) of pharmacists; (barrier or facilitator)<br>• Pharmacists' confidence in GP prescribing (i.e. not questioning the GP's prescription) (barrier)<br>• GPs feeling comfortable about their role in relation to specialists (facilitator)<br>• lack of resources and time (barrier)<br>• GPs' and pharmacists' beliefs about the complementary role of home care nurses (facilitator)<br>• Lack of formalization of the roles of each in the patient's pathway (lack of consideration of the pharmacist's role by GPs; difficulties in delegating) (barrier)	"...we are actually coordinating physicians for a little bit of everything, so we are there to make the link between all the specialists and all the other physicians who work with our patients and to see if the prescriptions are appropriate. Sometimes our colleagues don't have access to all the elements in the patient's file, so yes, yes, it's our role to make the link between everything and from time to time to modify the therapies that have been over-prescribed". (MG2)<br>"...No, I would say no, there's no reason for us to take, pardon the expression, the responsibility. it's everyone who has to take into account what the patient already has as treatment. Nothing prevents a psychiatrist or a gastroenterologist from asking the patient to show him his prescription. I don't see why we should centralize, insure, endorse." (MG1)<br>"...Since in theory yes I can have knowledge of his history of what he is taking ... To centralize a little bit of information from different practitioners.... But I only put myself in the place of a link like the pharmacist, the nurses who can intervene at home and see the use of other medications etc... "(MG5)<br>"...I see us as a second protection, that is to say that the doctor is already doing a job and we are really there to avoid all the mistakes..." (PH4)<br>"...The pharmacist can have an overall view since a given patient will go to the same pharmacy for supplies, so he can find in his history the different prescriptions of several prescribers, but we on the other hand sometimes don't even have a notion of what is prescribed elsewhere... the pharmacist as I said has a hindsight that the doctor doesn't have on prescriptions from different sources... the interaction and the reactivity of the pharmacist with the attending physician is important. " (MG1)<br>"...yes I find that pharmacists are not sufficiently involved in this...Communication and a kind of double reading of prescriptions that is not done sufficiently. "(MG3)<br>"...no, but because that's what we are talking about, these are things that we can't understand, for example when you have a Flecaine and a Bisoprolol, we can't do anything, we are obliged to trust the doctor who prescribed it, it's logical, it's his job... So we are not doctors, we don't know why all the drugs were prescribed... Anyway, when a drug is prescribed, if we respect the law, we are not supposed to know why it is prescribed... If it remains in normal posologies, in normal indications when the person explains to us why he is taking it; there is no reason to panic about it! After that, it's the responsibility of the doctor who prescribes it, that's all.“ (PH3)<br>"...correct yes, if ever I have a doubt or if ever I don't understand the indication I don't hesitate to call my colleague to try to understand the indication and to discuss it or to write him a letter and send the patient back to the colleague to discuss it... very regularly my colleagues suggest therapeutic modifications in relation to what they have seen and afterwards it's up to me to judge the necessity etc." (MG2)<br>"...No, it's not beyond our responsibility, of course it's time-consuming, etc. Afterwards, sometimes when we don't know, we call our cardiologist colleague, etc., and that helps us a lot, because we are general practitioners, we are not cardiologists, not neurologists, and there are things that we don't know, but we get help when we have questions. (you consider that it is your role to centralize all that?) yes yes because they only look at one organ, they only look at their own thing, they are not going to start looking at everything, that is logical. "(MG6)<br>"...we could, I agree with you, but it's not the same job anymore, it's not the same thing, today I would need three more pharmacists to do that ...It's not even a question of motivation, it's a question of changing the job. It's not even a question of motivation, it's a question of changing the profession. That is to say that today's pharmacies are not made for that, tomorrow we need waiting rooms, we need individual interviews with people, we need space... and so on. Today, no pharmacy, except when we were doing a little bit of pharmaceutical interviewing, has a business model that relies on that, it's not possible. It wouldn't be the same business anymore. " (PH3)<br>"...it's beyond our role that's what it is. That is to say, we don't have the means for this policy. "(MG1)<br>"...No (we don't have the conditions to fulfill this role), it's always the same problem; we don't have the time and we have too many things to manage at the same time.” (MG6)<br>"...maybe nurses would have a little more leverage than pharmacists, for people in the home, it's a caregiver who is in contact with the patients on a daily basis who know them; maybe they would have a little more leverage..." (MG6)<br>"...Afterwards, we must also see that in rural areas, when the person has major difficulties anyway, the role of the nurses is essential, because they manage all that (proper understanding of the treatment)" (PH3)<br>"There is a problem of pride among doctors, which means that they don't really want us to delegate work to them (...) ah well yes, if we had a little more esteem in the eyes of the doctors, it might be a little different, yes, that's clear (PH4)

TDF domains	Determinants (barriers and/or facilitators)	Illustrative quotes translated into English
**Belief about capabilities**	• Confidence or lack of confidence in the training received (barrier or facilitator)<br>• Pharmacists' awareness of the limits of their competence in analysing prescriptions (need to refer to GPs) (facilitator)<br>• Belief in the support of software (as a complement to training) (facilitator)<br>• The experience (facilitator)<br>• Knowing the patient (see Skills/ Trusting relationship GPS,Pharmacist/Patient: relational aspect, interactions, explanations, shared decision) (facilitator)<br>• Lack of access to the whole information in the patient file (barrier)<br>• Lack of resources and time (barrier)	"...basically I have everything in my head (laughs)" (MG4)<br>"I think as a general practitioner I'm the most competent person to handle this. Much more than the specialists... "(MG3)<br>"Already we're not trained for this. " (PH3)<br>"Not trained yes, after that we learn on the job...Maybe strengthen the knowledge in pharmacology and after on the psychological approach yes." (MG5)<br>"of course when it's something we can't handle ourselves... at the slightest doubt, when we're not sure of ourselves of course we call the doctor. It will depend on the medication, there are some things where it is less embarrassing than others. But for cardio drugs where there are fairly low doses, narrow therapeutic margins or things like that, it's better to have the doctor's opinion to be sure that there are no problems." (PH3)<br>"I have my Vidal database in my business tool... I use the Vidal yes automatically...it's enough for me yes, the Vidal and my continuing education. "(MG3)<br>"Now all pharmacies are equipped with software that will alert us to formal contraindications, even after there are precautions for use, and so on. "(PH3)<br>"If you follow the law, you're on the phone all the time calling a doctor, and after a while you have to know how to dial, with what you have, the questions you may have, the experience you have... " (PH3)<br>"Not trained yes, afterwards you learn on the job..." (MG5)<br>"it's seeing people again and being used to my patients that allows you to have a good critique in my opinion. ..." (MG4)
**Belief about consequences**	• Constant concern to avoid iatrogeny: drug interactions and side effects (facilitator)<br>• Notion of short- and long-term consequences (e.g. falls, hospitalizations, deterioration of general condition and domino effect) (facilitator)<br>• Belief that polypharmacy goes hand in hand with polypathology (barrier or facilitator)<br>• Belief in the impact on drug compliance (barrier or facilitator)	"...oh well it's to avoid all the adverse effects of treatments and then to justify treatments because we're not here to throw treatments around like that. ." (MG2)<br>"So Drug interaction and adverse effects that's it. In the medium and long term and the need to re-evaluate this as often as possible to avoid as much iatrogeny as possible. "(MG3)<br>"...it's the domino effect actually. Grandma breaks her hip in the night because she got up and has low blood pressure and at the same time she is completely groggy from her Lexomil and as a result she either breaks her hip and loses all her autonomy and the problems that follow. or she has a cerebral hematoma and so on ... and the cascade that follows" (MG6)<br>"...I am careful indeed not to make ... "big prescriptions" but I have a lot of heavy cardiovascular patients and no yes there are a lot of treatments." (MG7)<br>"...there's everything. Some have 2 or 3. Some may have fifteen or so. It will depend on the pathologies. No, generally speaking, the doctors try to limit it as much as possible, to reduce it, but sometimes you can't do otherwise anyway. On average 7 or 8" (PH3)<br>"I work on the principle that the less they have, the better; when we can, of course, sometimes we have large prescriptions and they need everything, but when we can like that, yes, it avoids problems with taking the medication, making mistakes, etc." (MG6)<br>"...Disadvantages, yes, notably the confidence in the medication that the patient can have, things like that; which is a very important guarantee of compliance. Yes, yes, I sometimes ask myself the question, re-evaluate and even change my decision if I have to." (MG3)

TDF domains	Determinants (barriers and/or facilitators)	Illustrative quotes translated into English
**Motivations and goals**	• Benefit-risk balance (facilitator)<br>• Tiredness; time of day or week (barrier)<br>• Lack of resources and time (barrier)<br>• Lack of access to the whole information in the patient file (barrier)<br>• Patient condition (age, chronicity, ability to understand/manage one' s treatment) (barrier/ facilitator)<br>• Patient profile: reluctant patient (barrier)<br>• High motivation to check the medication of the elderly (facilitator)<br>• Employing strategies: deferring, explaining, negotiating (facilitator)<br>• Being a training supervisor (facilitator)<br>• Habit/ chronic patients/ prescription refills (barrier)	"So we talked about it, and then we came to the conclusion that we should stop his treatment so that it wouldn't do him more harm than good." (MG2)<br>"Fatigue at some point, at the end of the week on a Friday evening, I will probably have less energy to fight than I would on Monday morning at 8:00 a.m., that's for sure." (MG6)<br>"...clearly sometimes the lack of time. we say to ourselves "well now I'm running late and then to explain to the patient that there won't be any major side effects etc..." sometimes it can happen, I mark it on the file and the next time I modify it." (MG2)<br>"...Afterwards, it's when I feel like I don't know all the patient's history, that I know everything, that I understand everything in the patient's file, so I say to myself that it's better to leave it for another three months than to stop it and that, in the end, we end up causing a problem by stopping it." (MG5)<br>"...Then it's the patient's condition, that is to say if there has ever been a major concern about changing the treatment, we try not to do everything at once. It also depends on the patient's age, if we know that he is unfortunately at the end of his life and that there is no indication to change him in the long term, if he doesn't want to, it won't change much, so here we are adapting the therapies in relation to that." (MG2)<br>"...Yes, of course, in fact I often try, I try anyway often, I try by discussing a little I gauge but here I am, I always try but it's true that I'm not going to exhaust myself doing it either; I try, it passes so much the better, it doesn't pass "so much the worse" (MG6).<br>"...I am not a die-hard. But at each consultation I discuss the usefulness of a medication" (MG4)<br>"... if I can't deal with it the first time I see a patient and I see things; except of course if I see a danger or an aberration that I'll deal with it right away, otherwise I'll try to discuss it again to explain why I'm questioning a treatment they've had for ten years..." (MG5)<br>"...I have the same motivation every time and what allows me to limit this pitfall is that it can happen especially with chronic patients that we see regularly, and what allows me to limit this is the intervention of the intern who is there every week and who allows me to re-evaluate everything. The intern allows me to ask myself questions and it is sometimes the intern who even suggests the question of the interest of a medication. Because the risk with habit is that it can limit the questioning." (MG3)

TDF domains	Determinants (barriers and/or facilitators)	Illustrative quotes translated into English
**Memory, attention and decision processes**	• Habit/ chronic patients/ prescription refills (barrier)<br>• Lack of time (barrier)<br>• Being a training supervisor (facilitator)<br>• Reducing prescription renewal intervals (facilitator)<br>• Deferring to better assess the case (facilitator) (see Motivations and goal/ Employing strategies: deferring, explaining, negotiating)<br>• Lack of access to the whole information in the patient file (barrier)<br>• Verification stage before printing the prescription by the GP - double check by the pharmacist when dispensing (facilitator)	"Time is a negative variable on treatment re-evaluation that's for sure..." (MG3)<br>"First I was in hospital internships we always found ourselves with his prescriptions that were ten pages long, I said to myself it's not possible! I didn't understand and then it was when I went to internship (at the GP) that I understood; for sure there is no time." ...<br>"Yes, yes, with an intern too, yes, that's two brains. There are three doctors in the practice and it's true that sometimes I call on them. It's certainly convenient, sometimes we're there and we leave the consultation to ask, we call each other "tell me what you think" ... Yes, yes, the specialists are quite approachable and available and sometimes they call back, yes, frankly... "(MG6)<br>"...I have very good relations with the pharmacists and nurses and we talk a lot, there are many exchanges... More than just having check-ups like that. A verbal discussion and then that's it...no need to do anything more complicated. " (MG4)<br>"There are doctors with whom it would go very well and doctors who would not have the time. There are many doctors who think they are the best, that is to say, even when we call them when there is a shortage of medication to find out what to put in its place and they send us packing." (PH4)<br>"It's true that having an intern allows us to question ourselves and then there are two of us; two for a consultation it goes much faster so we try. After that, what else could help me? apart from a human being with knowledge, I don't see ..." (MG6) (MG6)<br>"The pharmacist can have an overall view because a given patient will go to the same pharmacy for supplies, so he can find in his history the different prescriptions of several prescribers, but we, on the other hand, sometimes don't even have the notion of what is prescribed elsewhere." (MG1)<br>"Well, today they don't have the time and neither do I. They (the GPs) don't have the time. They (the general practitioners) are part of a medical centre, I think they have never had a meeting. I'm not part of it because pharmacists are not allowed. Afterwards, we can make projects or things like that... but today neither they nor I have the time to spend 2 hours in a meeting to discuss this. That doesn't mean that we won't do it on the phone or when we meet each other, but from there to formalize things I don't think..."(PH3)
**Social influences**	• The feeling of being completely free in prescribing and dispensing (facilitator)<br>• Anti-lab generation of GPs (facilitator)<br>• Regular training and information (e.g. via the journal Prescrire) to free oneself from any influence (facilitator)<br>• Financial aspect for pharmacists (barrier)<br>• Pressure from patient and/or family (barrier)<br>• Incentives/recommendations for non-prescription from the CPAM (facilitator)	"I read and subscribe to the journal Prescrire. Then, I don't receive medical visitors at the office and don't respond to their requests. I always try to remember to avoid adding a drug to treat a symptom... After that, I try to take training courses to keep up to date. But it's true that Prescrire remains a good source. "(MG5)<br>"Afterwards, when it comes to laboratories, we know that we should give more to one laboratory than to another because we get more discounts on certain laboratories...; afterwards, there is the financial aspect behind it, of course. Afterwards, a laboratory is generally worth as much as another one, so there are not too many problems. Overall, I feel quite free, but then obviously there is the financial aspect which is very important. "(PH4)<br>"Often it's the APs who have nothing to do all day who are the most stressed, who want things to go fast and to be served quickly, and that puts extra pressure on us. "(PH4)<br>"On the other hand also the difficulty of reducing treatments in a context let's say where we often have to account either to the families or other and not only to the AP. " (MG1)<br>"The only non-prescription incentive that exists; there are two in fact, it is the non-prescription incentive for antibiotics and that for long-acting Benzodiazepines. (MG3)
**Emotion**	• Patient profile: anxiety patients and psychiatric profiles (barrier)<br>• HCP's Tiredness; time of day or week (barrier)<br>• HCPs' confidence in their ability to cope with stress (facilitator)<br>• Employing strategies: deferring, explaining, negotiating (facilitator)	"People's stress or anxiety, psychiatric conditions, it's pretty hard to take the medication away. I talked about cardiovascular pathologies I can manage well but psychiatric problems it's more difficult; so there it's more complicated." (MG4)<br>"... It's true that fatigue at some point, at the end of the week on a Friday night, I'll probably have less energy to fight than on Monday morning at 8:00 a.m., that's for sure!" (MG6)<br>"No, logically no. It may influence something else, but I refuse to miss out on something. Afterwards, I'm not going to say that subconsciously I'm not going to do it, but no, no, you have to know how to separate things." (PH3)<br>"No, no, afterwards I am stressed like everyone else, we control it at certain times but no, no, in principle it does not affect me" (MG3)<br>"to talk with people, to explain, the why of the how. We can do it, but psychotic or neurotic people are more complicated" (MG4)<br>"when they become fixated on it, but we manage. It's true that there are some who come like that. But we explain to them why and in the vast majority of cases there is no problem." (PH3)

TDF domains	Determinants (barriers and/or facilitators)	Illustrative quotes translated into English
**Emotion**	• Patient profile: anxiety patients and psychiatric profiles (barrier)<br>• HCP's Tiredness; time of day or week (barrier)<br>• HCPs' confidence in their ability to cope with stress (facilitator)<br>• Employing strategies: deferring, explaining, negotiating (facilitator)	"People's stress or anxiety, psychiatric conditions, it's pretty hard to take the medication away. I talked about cardiovascular pathologies I can manage well but psychiatric problems it's more difficult; so there it's more complicated." (MG4)<br>"... It's true that fatigue at some point, at the end of the week on a Friday night, I'll probably have less energy to fight than on Monday morning at 8:00 a.m., that's for sure!" (MG6)<br>"No, logically no. It may influence something else, but I refuse to miss out on something. Afterwards, I'm not going to say that subconsciously I'm not going to do it, but no, no, you have to know how to separate things." (PH3)<br>"No, no, afterwards I am stressed like everyone else, we control it at certain times but no, no, in principle it does not affect me" (MG3)<br>"to talk with people, to explain, the why of the how. We can do it, but psychotic or neurotic people are more complicated" (MG4)<br>"when they become fixated on it, but we manage. It's true that there are some who come like that. But we explain to them why and in the vast majority of cases there is no problem." (PH3)
**Behavioral regulation**	• Employing strategies: deferring, explaining, negotiating (facilitator)<br>• Habit/ chronic patients/ prescription refills (barrier)<br>• High motivation to systematically check the medication of the elderly (facilitator)<br>• Lack of access to the whole information in the patient file (barrier)<br>• Patient profile: reluctant patient; patients attached to their treatment, ability to understand/manage one' s treatment) (barrier/ facilitator)<br>• Lack of resources and time (barrier)<br>• More or less effective communication between HCPs (barrier or facilitator)	"Afterwards, it's when I feel like I don't know everything about the patient's history, I don't know everything about the patient's file, so I think it's better to leave it for another three months than to stop it and end up causing a problem by stopping it." (MG5)<br>"For example, for this type of prescription (Mrs. Z), is it a renewal and therefore the risk is that we renew it without even asking the question of a possible interaction, or is it a one-time treatment and then, of course, we use the software to prescribe over it, if I may say so. The problem is that sometimes they are renewals and we always do the same thing over and over again without using the prescription analysis tool…" (MG1).<br>"Well, every time I write a prescription, a prescription renewal, I am very critical about everything I give. Depending on the general state, the state of the kidneys and the usefulness of the medication…" (MG4)<br>"It's about trying to convince the patient, especially because otherwise it's things we all know, but they've reached an age where they're sure they need their ten medications…" (MG6)<br>"In practice, except for doing this in a more specific setting where you have an hour to analyze a prescription with all the literature... there are people at 90 years of age who are perfectly managed, there are others at 40 years of age who can't manage it I mean... so it's more in relation to that that we have to be vigilant, it's not the fact that it's an elderly person or not, except if there are confusional states where we know that or if there is an onset of dementia, we know that we see them before the doctors do, so it's not unusual for us to be the ones to warn the doctors when there are errors…" (PH3)<br>"Sometimes when it's medication that's a bit out of the ordinary, we ask around a bit more, we take our time and then if we really have a doubt we call the doctors to communicate with them directly. Sometimes we discuss a treatment with them... it can happen (...) We may call the doctor to ask him why he put this on, or why we wouldn't put this on instead... and sometimes the doctor says to me "well yes, it's true, take it off, it's no use" (PH4)
**Nature of the behaviors**	• Verification stage before printing the prescription by the GP - double check by the pharmacist when dispensing (facilitator)<br>• Benefit-risk balance (facilitator)<br>• Prescribing based on recommendations and other checklists (facilitator)<br>• Using databases and software (facilitator)<br>• Relatively limited access to indicators and statistics (barrier)	"The more lines there are, the more attention we pay to the dosage, in pediatrics we also pay attention to everything, there are not times when we allow ourselves to dispense without looking. We have a double check on all prescriptions" (PH3) "after me it's particular because it's my thesis subject so I'm immersed in it and my checklist is in my head; for the moment it's easy...because I'm immersed in it, it gives me red flags. ." (MG6)<br>"I try to look at the new recommendations regularly...I have a software program with the old prescriptions in memory. Afterwards, in relation to the recommendations... ." (MG2)<br>"I have a software program with the old prescriptions in memory. Afterwards, the CPAM sends us data on the use of benzodiazepines and antibiotics at the end of the year and that's all..." (MG5)<br>"No I don't. Me: and you wouldn't be interested? MG4: yes yes yes, but there is a person from the social security who comes twice a year and shows me the statistics. Me: I have the impression that in your case, you have a little bit of everything in your head...am I wrong? MG4: yes, we know globally if we are doing things rather well or rather not." (MG4)
